# Therapeutic Pearls at Random Strung

**Published:** 1881-08

**Authors:** 


### THERAPEUTIC PEARLS AT RANDOM STRUNG.

### Neuralgia.

℞ Ext. hyosciamus.
   " valerian.
   Oxide zinc, . . āā ʒ i. Mix and make 60 pills.
   S.—One pill every three hours.

### Neuralgia and Rheumatism (Dr. Baltzell's ℞).

℞ Tinct. iodine comp.

   Aquæ ammonia, . . . āā ℥ iss.

   Pulv. camphor, . . . ʒ ij.

   Chloroform, . . . . ℥ ss. M.

   Rubbed in three or four times a day.

### Sore Nipples.

Dr. Barker, of New York, regards a solution of *nitrate of lead* in *glycerine*—10 grains of the former to an ounce of the latter—after a poultice has been applied to sore nipples, almost a specific. He also recommends it as a prophylactic against the occurrence of sore nipples in the highest terms.—*Obstetrical Journal* of Great Britain and Ireland, Oct. No , 1873. American Supplement, page 108.

### IODOFORM FOR SORE NIPPLES.

Nothing we have ever tried for the cure of sore nipples has done so much good as the following ointment: ℞ Iodoform ʒ ss-j; Lard, Simple Cerate, āā ʒ ij.—M. Apply it as often as the child is taken from the breast. Before suckling, wash the nipples thoroughly with soap and water. Great improvement may be expected in twelve hours from the time of the first application. The iodoform fills the house with its unpleasant odor, but "that is nothing compared with the sweet relief the medicine affords."

### NEURALGIA AND RHEUMATISM.

℞ Chloroform.

Tinct. aconite root.

Spts. tenbrith.

Tinct. opium, . . . āā f. ℥ ss.

Soap liniment, . . . f. ℥ iii to ℥ iv. M.

Apply locally on the seat of pain.

### TREATMENT OF HŒMORRHOIDS.

(Dr. Sabal, of Jacksonville, Florida.)

℞ Iodoform, . . . . . . ʒ i.
Powder very fine in a mortar, and add
Pulv. Opium, . . . . . grs. xv.
Pomade Vaseline (or Casmolin), . . ℥ i. Mix.

Apply locally, morning and night, and after every action of the bowels, after washing with warm or cold water. A drachm of tannin may be added to the above, if necessary. Keep the bowels open with Prof. Elliott's formula, viz:

℞ Magnesia Sulph.
Magnesia Carb.
Sulphuris Precipitat.
Sacchar. Lactis, . . . . . āā ℥ ss.
Pulv. Anisi, . . . . . ʒ ij. Mix.

Dose—One or two teaspoonfuls mixed in water at bedtime.

